# Lower long-term mortality in obese patients with community-acquired pneumonia: possible role of CRP

**DOI:** 10.6061/clinics/2019/e608

**Published:** 2019-07-03

**Authors:** Jin Chen, Jia Wang, Hui Jiang, Mao-Chun Li, Si-Yuan He, Xiao-Peng Li, Dantong Shen

**Affiliations:** I Department of Critical Care Medicine, Xiehe Hospital of Dongxihu District, Wuhan People's Hospital of Dongxihu District, Wuhan, 430000, China; II Department of Respiratory, Wanbei Coal-Electricity Group General Hospital, Suzhou 234011, Anhui Province, China; III Department of Pharmacy, The Central Hospital of Wuhan affiliated to Tongji Medical College, Huazhong University of Science and Technology, 430024, Wuhan, China; IV Department of Nephrology, Tongji Hospital affiliated to Huazhong University of Science and Technology, Wuhan, 430030, China; V Department of Neurosurgery, Tongji Hospital affiliated to Huazhong University of Science and Technology, Wuhan, 430030, China; VI Department of Neurologic Rehabilitation, Neurologic Specialized Hospital, General Hospital of Southern Theater Command, Guangzhou, 510010, China

**Keywords:** Obesity, Community-Acquired Pneumonia, Mortality, C-reactive Protein

## Abstract

**OBJECTIVE:**

The present study aimed to investigate the relationship between obesity and mortality in patients with community-acquired pneumonia (CAP) in China.

**METHODS:**

In total, 909 patients with CAP were recruited for this study from January 2010 to June 2015. All patients were selected and divided into 4 groups according to their body mass index (BMI) values. All patients' clinical information was recorded. The associations among mortality; BMI; the 30-day, 6-month and 1-year survival rates for different BMI classes; the etiology of pneumonia in each BMI group; and the risk factors for 1-year mortality in CAP patients were analyzed.

**RESULT:**

With the exception of the level of C-reactive protein (CRP), no other clinical indexes showed significant differences among the different BMI groups. No significant differences were observed among all groups in terms of the 30-d and 6-month mortality rates (*p*>0.05). There was a significantly lower risk of 1-year mortality in the obese group than in the nonobese group, (*p*<0.05). Logistic regression analysis showed that there were seven independent risk factors for 1-year mortality in CAP patients, namely, age, cardiovascular disease, cerebrovascular disease, obesity, APACHE II score, level of CRP and CAP severity.

**CONCLUSION:**

Compared with nonobese patients with CAP, obese CAP patients may have a lower mortality rate, especially with regard to 1-year mortality, and CRP may be associated with the lower mortality rate in obese individuals than in nonobese individuals.

## INTRODUCTION

Community-acquired pneumonia (CAP) is a common disease that frequently causes hospitalization and death in the elderly 1. As the 2010 Global Burden of Disease Study reported, lower respiratory tract infections, including pneumonia, have become the fourth most common cause of death worldwide, exceeded only by ischemic heart disease, stroke and chronic obstructive pulmonary disease (COPD) 2. Studies have already demonstrated several relevant risk factors for CAP, such as age >65 years, smoking, alcoholism, weight loss, immunosuppressive conditions, cardiovascular disease, poor functional and nutritional statuses, heart disease, renal disease, cerebrovascular disease and chronic obstructive pulmonary disease (COPD) 3. Additionally, environmental exposure to substances such as second-hand smoke, gases, fumes and chemicals also may lead to CAP 4.

Compared with younger age groups, the elderly have significant differences in terms of their demographics, risk factors, clinical presentations, and disease etiologies 5. Although there is no information about the microbial etiology of CAP in the current clinical guidelines, *Streptococcus pneumonia*, *Staphylococcus aureus* and *Enterobacteriaceae* are considered the main causes of pneumonia in the elderly; some previous studies also found that *Chlamydophila pneumoniae* and viruses can cause pneumonia in the elderly 6. Due to special conditions pertaining to elderly individuals, elderly patients with CAP always have poor prognoses, with a high mortality rate of approximately 5-15% 7; this high mortality rate remains a challenge in the clinical setting.

Obesity is generally considered a major risk factor for many diseases, such as hypertension, cardiovascular disease, lipid disorders, and type 2 diabetes 8. However, it is thought that patients a higher body mass index (BMI) values may have lower mortality rates in epidemiological studies 9. Recently, it was found that the mortality due to CAP is lower in obese elderly patients than in normal weight elderly CAP patients 10,11. However, due to a lack of adequate data, the mechanism underlying this difference is not clear, and the relationship remains contentious.

To the best of our knowledge, few studies have focused on the relationship between obesity and long-term mortality. The present study aimed to explore the impact of obesity on long-term mortality in CAP patients older than 55 years, as well as the possible mechanisms underlying this effect. This study may provide more clinical evidence and a better understanding of lower mortality in obese patients than in nonobese patients with CAP.

## METHODS

### Patients

In the present study, a retrospective analysis was conducted with 909 of the 1013 patients older than 55 years with CAP who presented at Wuhan People's Hospital of Dongxihu District, China, from January 2010 to June 2015. The diagnostic criteria for CAP were as follows: 1) fever (>38°C), cough, purulent sputum or change in the characteristics of the respiratory secretions; 2) a radiographic infiltrate compatible with pneumonia; and 3) laboratory data, such as leucopenia, leukocytosis, and increased arterial-alveolar gradient 6. Patients with immunosuppression, organ transplantation, active thoracic malignancy, hospital-acquired pneumonia, pulmonary embolism, chemotherapy, or corticosteroid therapy were excluded. All patients were treated according to the local management protocols for CAP. Patients were divided into different groups according to body mass index (BMI) values as follows: 1) obese (BMI >30), 2) overweight (BMI 25 to <30), 3) normal (BMI 18.5 to <25), and 4) underweight (BMI<18.5). The study was approved by the Ethics Committee of Wuhan People's Hospital of Dongxihu District.

### Data collection and patient assessment

All patients underwent clinical and laboratory evaluations. Baseline observations (including blood pressure and respiratory frequency) and standard blood tests (complete blood count, liver function tests, coagulation profile, white blood cell count (WBC), and the determination of the levels of urea, electrolytes, C-reactive protein (CRP), procalcitonin (PCT), interleukin-1 (IL-1) and tumor necrosis factor (TNF-α)) were performed upon patient admission. Biomarker levels were measured within 24h after admission.

Acute Physiology and Chronic Health Enquiry (APACHE-II) scores and the Pneumonia Severity Index (PSI) were used to assess the risk of CAP in the patients 12,13.

Of the initial 1013 patients, 104 patients were excluded, and 909 (89.73%) patients were included. These patients had a known 1-year survival status and BMI. We also recorded the 30-day survival, 6-month survival and 1-year survival for different BMI classes to determine the relationship between BMI and mortality. All patients were followed for 1 year by telephone or outpatient follow-up visits. Furthermore, the etiology of pneumonia was also studied according to BMI group was in this report. The detailed clinical information for all patients is listed in Table 1.

### Statistical analysis

Patient characteristics are presented as medians (IQR 5^th^-95^th^ percentiles), and frequencies are expressed as percentages. Comparisons between two groups were performed with Student's t-test, and comparisons among three or more groups were conducted using one-way analysis of variance (ANOVA) followed by the Tukey post hoc test. The Chi-square test was used to compare the rates. For the survival analysis, survival time was calculated from the date of hospital discharge to the date of death up to 1 year, and Kaplan–Meier curves were generated. The association between mortality and BMI class was further assessed by Cox regression analysis adjusted for the PSI scores (model 1=adjusted for severity), sex, age, APACHE II scores, cardiovascular diseases, and other comorbidities including biomarkers (model 2=fully adjusted) with the normal BMI group (18.5 to 15 kg/m^2^) defined as the reference group. Moreover, hazard ratios (HRs) with 95% confidence intervals (CIs) were used to present the results of the time to the event analysis. For logistic analysis, univariate analysis was performed using χ^2^ tests and Student's t-tests as described above, and the significant factors in the univariate analysis were included in logistic multivariable regression to define possible risk factors for 1-year mortality in patients. Multivariable analysis was performed with logistic regression models with a stepwise adjustment method.

All calculations were performed with SPSS version 18.0 (SPSS Inc., Chicago, USA). A *p-*value <0.05 was considered statistically significant.

## RESULTS

### Basic clinical information for all included patients

According to the BMI levels, out of the total 909 CAP patients older than 55 years old, 73 (7.7%) were in the underweight group, 409 (45.3%) were in the normal weight group, 285 (31.4%) were in the overweight group, and 142 (15.6%) were in the obese group. Clinical characteristics, symptoms, signs and laboratory test results were recorded on admission, and the patients were classified by BMI. The results are shown in Table 1. The median duration of hospital stay for the study groups was 5 days (IQR 3-13 days), and the longest mean duration was for the underweight group, followed by the obese group. However, there were no significant differences in the duration of hospital stay among the groups. Regarding the inflammatory factors, there were no significant differences between different groups in terms of the WBC count (*p*=0.162), PCT level (*p*=0.982), IL-1 level (*p*=0.658), or TNF-α level (*p*=0.102), but a significant difference was observed in the level of CRP in different groups (*p*=0.045).

### Association between mortality and BMI classes after 30 days, 6 m, and 1 year

**Figure 1:**
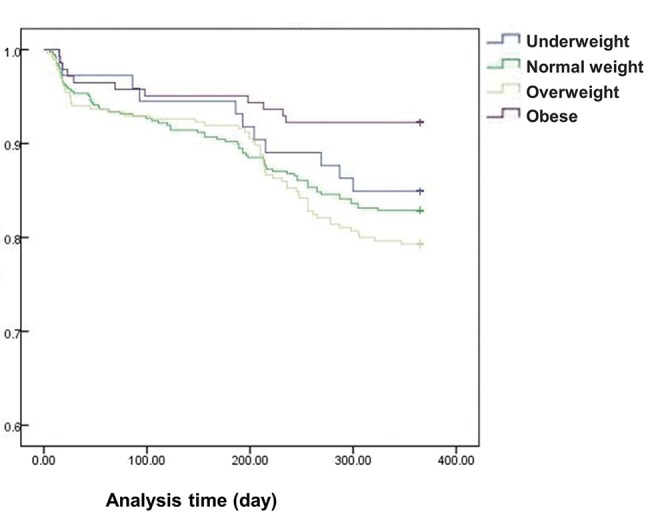
Kaplan–Meier curve of survival according to different body mass index.

In this study, we also followed patient mortality 30 days, 6 months and 1 year after their discharge from the hospital, and the mortality rates were 4.7% (95% CI 3.4-6.8), 8.6% (95% CI 8.3-11.9) and 14.9% (95% CI 12.4-18.2), respectively (Table 2). In this analysis, the normal weight group (BMI 18.5-25.0) was the reference group. Model 1 and model 2 represent different BMI groups. The association between higher BMI and 30-day mortality and 6-month mortality was not significantly different between the two models. However, mortality was significantly lower in the obese group than in the reference group in models 1 and 2 (*p*<0.05) (Table 2 and Figure 1). To confirm whether inflammatory factors can explain the reduced mortality in the obese group, these factors were also included in the fully adjusted model. Moreover, the effect of obesity on mortality was not changed by the inclusion of biomarker levels.

### Distribution of etiologies in different BMI classes

Finally, this paper studied the distribution of etiologies in different BMI classes (Table 3). The most frequent etiology in patients was infection with *S. pneumonia*, followed by pneumococcal bacteremia and infection with *Haemophilus influenza*. No significant differences were found among the four groups regarding the prevalence of pathogens.

### Risk factors for 1-year mortality in CAP patients

Finally, we investigated the risk factors for 1-year mortality in CAP patients. Risk factors associated with mortality in CAP patients were compared with those in living patients, and the results are shown in Table 4. Among the fifteen factors investigated, seven factors, namely, age, cardiovascular disease, cerebrovascular disease, obesity, APACHE II scores, and severity levels of CRP and CAP, were significantly different in deceased patients than in living patients (*p*<0.05). These factors were further analyzed by multivariable logistic regression analysis. The Hosmer-Lemeshow test showed that the model fit well (sig.=0.526). The results showed that all seven factors were associated with and independent risk factors for 1-year mortality in CAP patients (Table 5). These results were consistent with the results of the analysis of the relationship between obesity and 1-year mortality in CAP patients and thus further demonstrated the relationship between these factors.

## DISCUSSION

Community-acquired pneumonia (CAP) remains a leading infectious cause of morbidity and mortality 14, and it frequently causes hospitalization and death in the elderly. A range of lifestyle factors and underlying medical conditions, such as alcoholism, smoking, and chronic heart diseases, are associated with an increased risk of CAP 3. However, despite the substantial progress in diagnostic methods and treatment technology for CAP, the poor prognosis of CAP is still a large problem in the clinical setting.

Recently, the phenomenon that obese elderly patients with CAP might have a lower mortality rate than that of normal weight CAP patients has attracted scholars' attention. Several studies have reported related findings; however, a deeper understanding and more clinical evidence are still needed to demonstrate the relationship between obesity and lower mortality in elderly patients with CAP, especially with regard to the relationship between obesity and long-term mortality in CAP patients. In this report, we demonstrated that obese individuals might have a lower mortality rate than nonobese individuals, especially with regard to 1-year survival after admission, and we demonstrated that the effect might be associated with the level of CRP. We also demonstrated that obesity might be an independent risk factor for mortality in CAP patients, which was rarely seen in previous studies.

First, we found that, unlike the CRP level, the levels of PCT, TNF-α and IL-1 showed no significant difference in obese and nonobese CAP patients. Singanayagam et al demonstrated that compared with nonobese patients, obese patients had higher levels of C-reactive protein 7. Braun N et al found that the WBC counts and PCT levels did not vary significantly in different BMI groups 15. The results of these related studies are consistent with our findings.

Second, we found that elderly obese patients (BMI>30 kg/m^2^) with CAP had lower mortality rates than other groups of patients. However, the difference in the mortality rate was only found to be significant for the 1-year mortality rate, while the differences were not significant for the 30-day mortality and 6-month mortality rates in different BMI groups, indicating that the influence of obesity on mortality in CAP patients might be mainly long term. A previous report 15 found lower mortality rates occurred in the obese group than in the nonobese group after 6 years and also found no differences in the 30-day and 1-year mortality rates in different BMI groups. In addition, Hong et al demonstrated that frailty and readmission due to CAP were associated with higher 1-year mortality rates in older patients hospitalized for CAP 16. These different results might be due to different study populations and the different conditions of the patients, and they need further research for confirmation. The etiology of pneumonia was also investigated in our study. The distributions of risk factors were not significantly different in different BMI classes, which was similar to the findings of other related studies 17.

Finally, we investigated the possible risk factors for mortality in CAP patients, and the results showed that age, cardiovascular disease, cerebrovascular disease, obesity, APACHE II scores and CAP severity were all independent risk factors for 1-year mortality in CAP patients. Some related studies have identified the risk factors for mortality in CAP patients. Que et al studied CAP patients in the ICU and found that a CRP value at admission <169.5 mg/L predicted a fatal outcome 18. In a 5-year study of long-term mortality after hospitalization for CAP, the authors found that age, cardiovascular disease, COPD, immunocompromised status, and low serum albumin level at admission were all independent risk factors for long-term mortality in CAP patients 19. However, whether obesity is an independent risk factor for mortality in CAP patients or not remains controversial. The study also had some limitations. First, the study sample size was small. Second, the mechanism by which the level of CRP influences mortality in obese CAP patients is still unclear and needs further study.

In conclusion, the present study was a retrospective analysis of the relationship between obesity and lower mortality in patients >55 years with CAP. The results showed that the obese group had a lower 1-year mortality rate than the nonobese group, and the effect might be associated with CRP levels at admission. Moreover, both obesity and CRP levels were independent risk factors for 1-year mortality in CAP patients. These results may provide more clinical evidence and better understanding of reduced mortality in obese CAP patients.

## AUTHOR CONTRIBUTIONS

Chen J conducted most experiments and wrote the manuscript. Wang J contributed to the manuscript revision, language correction and statistical checking. Jiang H revised the manuscript, evaluated the study quality and designed the study steps. He SY and Li XP collected the data. Li MC revised the manuscript and analyzed the data. Shen D provided the funding support, revised the manuscript, evaluated the study quality and designed the study steps.

## Figures and Tables

**Table 1:** Patient characteristics categorized by BMI.

	Overall	Underweight (BMI <18.5)	Normal weight (BMI 18.5-25.0)	Overweight (BMI 25.0-30.0)	Obese (BMI >30)	*p-*value^a^
N	909	73 (7.7%)	409 (45.3%)	285 (31.4%)	142 (15.6%)	
Age (y), median (IQR)	72 (57-83)	70 (56-85)	74 ( 57-84)	75 (60-83)	69 (55-79)	0.211
Fever (>38°C), n (%)	633 (69.6)	50 (68.5)	286 (69.9)	205 (71.9)	92 (64.8)	0.438
Cough	335 (36.9)	33 (45.2)	143 (35.0)	108 (37.9)	51 (35.9)	0.604
Median duration of admission (d) , median (IQR)	5 (3-13)	7 (3-12)	5 (2-11)	5 (3-13)	6 (3-12)	0.185
Female, n (%)	431 (47.4)	32 (43.8)	213 (52.0)	123 (43.2)	63 (44.4)	0.560
Current smoker, n (%)	227 (25.0)	18 (24.7)	110 (26.9)	74 (26.0)	25 (17.6)	0.398
Cardiovascular disease, n (%)	261 (28.7)	13 (17.8)	110 (26.9)	76 (26.7)	32 (22.5)	0.380
Cerebrovascular disease, n (%)	103 (11.3)	8 (11.0)	43 (10.5)	32 (11.2)	20 (14.1)	0.857
Hypertension, n (%)	211 (23.2)	12 (16.4)	85 (20.8)	74 (26.0)	40 (28.2)	0.188
Type II diabetes, n (%)	185 (20.4)	9 (12.3)	76 (18.6)	65 (22.8)	35 (24.6)	0.128
Scores of APACHE II	18 (8∼31)	19 (9∼28)	17 (8∼31)	21 (8∼29)	18 (9∼28)	0.235
Laboratory findings at admission, median (IQR)						
WBC	12.2 (8.9-16.3)	13.8 (11.3-17.8)	12.5 (8.7-17.6)	12.3 (9.2-15.9)	11.4 (9.0-16.2)	0.162
CRP (mg/L)	154.4 (74.0-237.0)	132.1 (67.3-217.9)	151.6 (75.8-243.5)	157.3 (74.5-245.0)	189.3 (97.3-278.2)	0.045
PCT (ug/L)	0.52 (0.16-3.31)	0.61 (0.22-3.89)	0.54 (0.17-3.24)	0.59 (0.21-3.01)	0.41 (0.15-3.42)	0.982
TNF-α (pg/mL)	13.5 (11.2-20.5)	15.6 (13.43-21.6)	12.5 (10.33-17.03)	13.5 (11.42-21.34)	14.2 (11.8-19.8)	0.102
IL-1 (ng/L)	27.8 (13.5-30.9)	25.6 (12.3-30.2)	27.2 (13.6-29.9)	26.1 (12.9-31.4)	30.4 (16.7-34.8)	0.658
CAP severity, n (%)						
PSI grade 1	109 (12.0)	9 (12.3)	54 (13.2)	31 (10.9)	15 (10.6)	0.924
PSI grade 2	150 (16.5)	12 (16.4)	70 (17.1)	44 (15.4)	24 (16.9)	0.989
PSI grade 3	165 (18.2)	15 (20.5)	74 (18.1)	55 (19.3)	21 (14.8)	0.749
PSI grade 4	331 (36.4)	24 (32.9)	143 (35.0)	106 (37.1)	58 (40.8)	0.900
PSI grade 5	154 (16.9)	13 (17.8)	68 (16.6)	49 (17.2)	24 (16.9)	0.813
Mortality, n (%)						
30 d	43 (4.7)	2 (2.7)	19 (4.6)	17 (6.0)	5 (3.5)	0.676
6 m	76 (8.4)	4 (5.5)	40 (9.8)	25 (11.6)	7 (4.9)	0.350
1 yr	151 (14.9)	11 (15.1)	70 (17.1)	59 (20.7)	11 (7.7)	0.072

a Compared among the 4 groups.

**Table 2:** Association between mortality and BMI classes after 30 d, 6 m, and 1 yr.

Mean % (95% CI)	30 d 4.7 (3.4-6.8) HR adj. (95% CI)	*p*-value	6 m 8.6 (8.3-11.9) HR adj. (95% CI)	*p*-value	1 y 14.9 (12.4-18.2) HR adj. (95% CI)	*p*-value
Model 1: Severity adjusted						
Normal wright	Reference		Reference		Reference	
Underweight	0.534 (0.075-4.145	0.567	0.925 (0.388-2.250)	0.831	0.935 (0.580-1.604)	0.912
Overweight	1.201 (0.543-2.340)	0.609	0.915 (0.642-1.413)	0.765	0.813 (0.627-1.044)	0.087
Obese	0.790 (0.274-2.109)	0.653	0.504 (0.312-1.107)	0.055	0.650 (0.457-0.789	**0.007**
Model 2: Full adjusted						
Normal wright	Reference		Reference		Reference	
Underweight	0.450 (0.054-3.576)	0.417	1.101 (0.456-2.675)	0.754	1.002 (0.596-1.879)	0.825
Overweight	1.088 (0.521-2.165)	0.802	1.043 (0.702-1.615)	0.743	0.688 (0.596-1.022)	0.075
Obese	0.618 (0.217-1.879)	0.412	0.634 (0.410-1.201)	0.125	0.720 (0.501-0.954)	**0.034**

Note: Adjusted for PSI (Model 1), PSI/age/sex/ Scores of APACHE II /cardiovascular diseases /other comorbidities including biomarkers (Model 2).

**Table 3:** Etiology of Pneumonia by BMI group.

	Overall	Underweight (BMI <18.5) n (%)	Normal weight (BMI 18.5-25.0) n (%)	Overweight (BMI 25.0-30.0) n (%)	Obese (BMI >30) n (%)	*p*-value
	909	73 (7.7%)	409 (45.3%)	285 (31.4%)	142 (15.6%)	
*Streptococcus pneumoniae*	371 (40.8)	31 (49.3)	166 (40.6)	110 (38.6)	64 (41.5)	0.440
Pneumococcal bacteremia	113 (12.4)	11 (15.1)	51 (12.5)	36 (12.6)	15 (10.6)	0.809
*Haemophilus influenzae*	86 (9.5)	6 (8.2)	38 (9.3)	29 (10.2)	13 (9.2)	0.971
Aspiration pneumonia	87 (9.6)	7 (9.6)	44 (10.8)	27 (9.5)	9 (6.3)	0.717
*Legionella pneumophila*	78 (8.6)	5 (6.8)	31 (7.6)	27 (9.5)	15 (10.5)	0.777
*Staphylococcus aureus*	17 (1.9)	2 (2.7)	7 (1.7)	6 (2.1)	2 (1.4)	0.921
*Gram-negative bacilli*	36 (4.0)	3 (4.1)	18 (4.4)	12 (4.2)	3 (2.1)	0.808
*Pseudomonas aeruginosa*	40 (4.4)	2 (2.7)	17 (4.2)	13 (4.6)	8 (5.6)	0.786
*Atypical agents*	36 (4.0)	3 (4.1)	18 (4.4)	12 (4.2)	3 (2.1)	0.808
Other	45 (5.0)	3 (4.1)	19 (4.6)	13 (4.6)	10 (7.0)	0.787

**Table 4:** Risk factors associated with 1 year mortality of CAP patients compared with the survival patients.

	Live, n=758	Death, n=151	*p-*value
Age (y), median (IQR)	72 (55∼79)	79 (62∼84)	**0.046**
Female, n (%)	351 (46.3)	80 (53.0)	0.354
Current smoker, n (%)	192 (25.3)	32 (21.2)	0.490
Cardiovascular disease, n (%)	199 (26.2)	62 (41.1)	**0.026**
Obesity, n (%)	131 (17.3)	11 (7.3)	**0.031**
Cerebrovascular disease, n (%)	72 (9.2)	31 (20.5)	**0.025**
Hypertension, n (%)	171 (22.6)	40 (26.5)	0.522
Type II diabetes, n (%)	143 (18.9)	42 (27.8)	0.137
Scores of APACHE II	17 (8-29)	21 (13-31)	**0.005**
Laboratory findings at admission, median (IQR)			
WBC	12.6 (8.7∼15.9)	13.1 (8.9∼16.4)	0.561
CRP (mg/L)	157.5 (75.6∼262.8)	151.6 (71.5∼231.6)	**0.012**
PCT (ug/L)	0.51 (0.16∼3.39)	0.53 (0.18∼3.28)	0.457
TNF-α (pg/mL)	13.6 (11.4∼21.9)	13.9 (11.6∼22.0)	0.538
IL-1 (ng/L)	28.1 (12.5∼30.5)	28.6 (12.9∼32.1)	0.649
CAP severity, n (%)			
PSI grade 1	106 (14.0)	3 (2.0)	**0.000**
PSI grade 2	135 (17.8)	15 (9.9)	
PSI grade 3	144 (19.0)	21 (13.9)	
PSI grade 4	283 (37.3)	48 (31.8)	
PSI grade 5	90 (11.9)	64 (42.4)	

**Table 5:** Risk factors associated with 1 year mortality of CAP patients by Logistic multivariate regression analysis.

	Wald	Odds ratio	95% CI	*p-*value
Age	2.653	0.936	(0.061∼2.126)	0.006
Cardiovascular disease	13.447	1.973	(1.376∼2.835)	0.000
Cerebrovascular disease	5.735	3.428	(1.537∼9.825)	0.000
Obesity condition	1.456	1.556	(1.337∼2.537)	0.002
Level of CRP	4.369	2.213	(1.525∼4.136)	0.003
Scores of APACHE II	2.356	1.785	(1.467∼3.215)	0.000
CAP severity	7.674	5.963	(1.485∼8.214)	0.000
